# The Evolution of Esophagectomy to Robotic-Assisted Minimally Invasive Esophagectomy (RAMIE) in the Treatment of Esophageal Cancer: Historical Insights and Comparative Outcomes

**DOI:** 10.3390/cancers18152444

**Published:** 2026-07-29

**Authors:** Erin Hoover, Aitua Salami, Matthew Porembka, Inderpal Sarkaria

**Affiliations:** 1 Division of Thoracic Surgery, Department of Cardiovascular and Thoracic Surgery, University of Texas Southwestern Medical Center, Dallas, TX 75390, USA; erin.hoover@utsouthwestern.edu (E.H.); aitua.salami@utsouthwestern.edu (A.S.); 2 Division of Surgical Oncology, Department of Surgery, University of Texas Southwestern Medical Center, Dallas, TX 75390, USA; matthew.porembka@utsouthwestern.edu

**Keywords:** esophageal cancer, robotic-assisted minimally invasive esophagectomy (RAMIE), minimally invasive esophagectomy (MIE), open esophagectomy (OE), robotic surgery, esophagectomy, thoracic surgery, surgical oncology

## Abstract

Robotic-assisted minimally invasive esophagectomy (RAMIE) has emerged as an increasingly utilized approach for the surgical management of esophageal malignancies, offering potential advantages over conventional minimally invasive esophagectomy (MIE) and open esophagectomy (OE). Advances in robotic technology, enhanced visualization, instrument articulation, and surgeon ergonomics have facilitated wider adoption of RAMIE in thoracic surgical oncology. This review summarizes contemporary evidence regarding intraoperative, postoperative, oncologic, and patient-centered outcomes associated with RAMIE while highlighting emerging technologies and future directions in the field. Current literature, including randomized controlled trials, meta-analyses, propensity-matched studies, and international registry data, suggests that RAMIE is associated with reduced blood loss, improved lymph node harvest, and lower pulmonary morbidity compared with OE, while demonstrating outcomes comparable or favorable to conventional MIE. Oncologic outcomes, including R0 resection rates and overall survival, appear equivalent across approaches in most contemporary studies. In addition, robotic platforms may facilitate recurrent laryngeal nerve lymphadenectomy and complex mediastinal dissection. Despite these advantages, RAMIE is frequently associated with longer operative times and remains dependent on institutional expertise, surgeon experience, and resource availability. Current evidence is promising but remains limited by heterogeneity of surgical techniques, institutional expertise, and a relative scarcity of long-term randomized data. Emerging innovations including fluorescence-guided imaging, perfusion assessment technologies, artificial intelligence integration, augmented reality, and advanced robotic platforms may further enhance precision, safety, and personalization of esophageal cancer surgery. As robotic technology and perioperative systems continue to evolve, ongoing prospective investigation and long-term oncologic evaluation will remain essential to define the optimal role of RAMIE within multidisciplinary esophageal cancer care.

## Simple Summary

Surgery to remove cancer of the esophagus is one of the most complex operations performed and carries a substantial risk of complications. New robotic technology has been developed to improve the precision of this operation, but questions remain about whether it provides meaningful benefits over traditional open surgery or standard minimally invasive techniques. This review examines the history of esophageal surgery, summarizes the best available evidence comparing these surgical approaches, and discusses emerging technologies that may shape future care. Current research suggests that robotic surgery can reduce blood loss, improve removal of nearby lymph nodes important for cancer treatment, and lower lung complications while achieving cancer control and long-term survival comparable to other techniques. However, robotic surgery often requires longer operating times, specialized training, and greater financial investment. This review also highlights promising advances, including artificial intelligence, improved imaging during surgery, and next-generation robotic systems, which may further improve safety and precision. By summarizing current knowledge and future directions, this review aims to help surgeons, researchers, and health systems make informed decisions that improve outcomes and quality-of-life for people undergoing surgery for esophageal cancer.

## 1. Introduction

Esophageal cancer is the eighth most common cause of cancer worldwide. Global incidence of esophageal cancer continues to rise [1,2,3]. Within the United States, it is the sixth most common gastrointestinal malignancy, with more than 54,000 individuals currently living with the disease [4,5]. The annual incidence is 4.2 per 100,000 men and women, with a nearly equivalent mortality rate of 3.7 per 100,000 [5,6]. Globally, both incidence and mortality have risen by approximately 6% from 2022 to 2025 [7]. This high death rate reflects both the frequent detection of esophageal cancer at advanced stages and its highly aggressive biology, which is associated with rapid progression and poor prognosis even when identified early [6,7]. Globally, esophageal cancer ranks as the sixth most fatal cancer [7,8,9]. The overall five-year survival rate is 21.9%, though outcomes improve to nearly 50% with the addition of neoadjuvant chemotherapy [2,5,6]. A total of 0.5% of individuals will be diagnosed with esophageal cancer during their lifetime. Surgical resection remains the gold standard for oncologic treatment and for palliation of symptoms such as dysphagia [1,3,5,10,11]. Even with optimal patient selection, however, esophagectomy carries a morbidity rate of 30–60% [2]. The challenge of balancing effective oncologic control with reduced morbidity and mortality has been universally recognized.

Although numerous studies have compared robotic-assisted minimally invasive esophagectomy (RAMIE) with conventional minimally invasive and open esophagectomy, the literature has expanded rapidly in recent years with the publication of randomized controlled trials, large international registry studies, and multiple contemporary meta-analyses. Existing reviews frequently evaluate minimally invasive esophagectomy as a whole or focus on isolated technical aspects of robotic surgery, whereas few provide a comprehensive synthesis dedicated specifically to RAMIE across its historical evolution, contemporary comparative outcomes, current limitations, and emerging innovations. This narrative review therefore summarizes the evolution of RAMIE from its historical origins to current clinical practice, critically evaluates the highest-quality evidence regarding intraoperative, postoperative, oncologic, and patient-centered outcomes compared with conventional minimally invasive and open esophagectomy, and discusses current challenges, future technological advances, and the evolving role of RAMIE in multidisciplinary esophageal cancer care.

## 2. Methods

Data were drawn from a comprehensive literature search of PubMed and Scopus.

This narrative review synthesizes evidence from original retrospective or prospective cohort studies, database/registry studies, randomized controlled trials, and meta-analyses selected for inclusion by an expert panel. Studies were summarized descriptively without formal assessment of overlap among patient populations included in published meta-analyses. Accordingly, these analyses may incorporate many of the same primary studies and, consequently, overlapping patient cohorts. Therefore, concordant conclusions among meta-analyses may reflect shared underlying data rather than independent replication and should be interpreted with caution and within the context of the overall body of evidence. The currently available literature includes studies of varying methodological quality, levels of evidence, and geographically diverse patient populations.

## 3. Historical Insights

The history of esophageal surgery spans more than a century, beginning with Franz Torek’s first thoracic esophageal resection in 1913 and culminating in the advent of robotic-assisted minimally invasive esophagectomy (RAMIE) in the early 2000s [8,12]. Advances in neoadjuvant chemoradiation expanded the pool of patients eligible for resection, driving the evolution of surgical techniques toward safer and more feasible approaches. Torek’s original procedure involved a two-stage operation: left thoracotomy with esophageal resection, followed by extra-thoracic reconstruction to restore gastrointestinal continuity [12]. Although his prototypical patient survived for 12 years, similar operations were not repeated for two decades, and mortality rates remained prohibitively high at 70–90% [12]. Subsequent variations—including esophagostomy with gastric feeding access, extra-thoracic “skin tube” conduits, and pre-sternal jejunal or colonic interpositions—were attempted but carried similarly high mortality [12].

By the 1940s, Ivor Lewis described a two-stage approach—combining abdominal and right thoracic incisions—with pedicled gastric pull-through and intrathoracic anastomosis [11]. Subsequently, in 1976, McKeown introduced the three-field esophagectomy, incorporating a cervical anastomosis to address proximal and mid-esophageal tumors [13]. A major advance came in 1992 when Cuschieri introduced thoracoscopic esophageal mobilization, paving the way for hybrid and total minimally invasive esophagectomy (MIE) [11,14]. The primary goal of MIE was to reduce respiratory complications and overall morbidity, thereby facilitating quicker recovery. A decade later, Luketich and colleagues reported a landmark series of over 1000 patients undergoing total MIE, demonstrating low rates of pneumonia and 30-day mortality [15,16]. The Traditional Invasive versus Minimally Invasive Esophagectomy (TIME) randomized controlled trial subsequently confirmed the feasibility of MIE, showing comparable perioperative and oncologic outcomes to open esophagectomy (OE) [13].

These developments—along with the advent of state-of-the-art energy instruments and minimally invasive staplers, among other technological advances in the field of surgery—set the stage for robotic-assisted minimally invasive esophagectomy (RAMIE), offering solutions to the limitations of conventional MIE, which is now increasingly utilized. Horgan performed the first hybrid robotic transhiatal esophagectomy with cervical incision in 2003, followed by Kernstine’s McKeown robotic-assisted transthoracic esophagectomy in 2004 [10,11,17]. In 2013, Sarkaria et al. reported the first successful series of robotic-assisted Ivor Lewis esophagectomies with thoracic anastomosis [18,19]. By 2010, 9% of esophagectomies in the United States were performed robotically, a figure that rose to 27.2% by 2016 [20].

RAMIE gained traction as an alternative to conventional MIE because its technical advantages—enhanced maneuverability, tremor filtration, motion scaling, and advanced energy tools—facilitate precise dissection around delicate structures [21,22,23]. Beyond mechanical advantages, robotic platforms provide a three-dimensional, magnified, high-definition visual field, enhancing depth perception and visibility compared to conventional MIE [24,25]. Advanced imaging capabilities allow assessment of organ perfusion and identification of key anatomy using indocyanine green (ICG) fluorescence [8]. In more recent iterations, integrated machine-learning applications enable “force feedback,” helping to mitigate the loss of tactile sensation inherent to robotic surgery [11,25,26]. Finally, the surgeon console improves ergonomics and reduces fatigue, contributing to career longevity [6,21,22]. A large number of robotic surgical platforms are available globally with ever-increasing capabilities. Of note, the term “RAMIE” represents the use of a robotic platform to perform esophagectomy but does not specify whether a procedure is performed in a fully (abdominal and thoracic) minimally invasive approach versus a hybrid (combination of an open abdominal and robotic thoracic) approach. This broad review does not distinguish between hybrid versus totally robotic approaches for all outcomes.

## 4. Clinical Outcomes of RAMIE Versus Conventional MIE and OE

### 4.1. Intraoperative Metrics

Intraoperative metrics with literature comparisons include estimated blood loss, lymph node harvest, and operative time (Table 1).

#### 4.1.1. Blood Loss

RAMIE was initially reported to have similar operative blood loss as MIE and OE; however, more recent studies suggest RAMIE incurs less operative blood loss than both MIE and OE. Two comparative studies from 2012 found no significant difference in estimated blood loss (EBL) between RAMIE and MIE, while a pooled analysis published in 2020 also failed to identify a difference in EBL between robotic and thoracoscopic approaches to esophagectomy [42,52,53]. Recent high-quality studies demonstrate that RAMIE may be associated with significantly lower intraoperative blood loss compared with MIE [23]. However, evidence is not uniformly consistent. A systematic review, published in 2022, identified a significantly reduced EBL among RAMIE procedures with a median EBL of 209.59 cc versus 374.38 cc in OE (*p* < 0.001) with a weighted mean difference of −296.94 cc when performing RAMIE [33]. A meta-analysis, published in 2021, identified a significantly lower blood loss when comparing RAMIE to MIE (−35.15 cc with mean RAMIE EBL of 170 cc), although the effect was smaller than that of comparing RAMIE to OE [37]. The prospective, multi-center German da Vinci Xi Registry Trial similarly identified a median blood loss of 200 cc (range 80–400 cc) for RAMIE. A significant reduction in EBL was associated with passing an inflection point of 22 cases in the RAMIE learning curve [23]. Suggested etiologies of decreased EBL include diminished tissue trauma due to the minimally invasive approach. Of note, many confounding variables exist in regard to operative blood loss, particularly patient-related factors such as comorbidities, medication use, and prior cancer treatment. An overall reduction in EBL has been linked to decreased transfusion requirements, decreased postoperative atrial fibrillation related to depleted intravascular volume, and less postoperative complications [33]. Still, these findings suggest a minimal reduction in volume of blood loss—on the matter of cubic centimeters—which may not be entirely of clinical significance.

#### 4.1.2. Lymph Node Harvest

Lymph node (LN) retrieval is a surrogate marker for oncologic outcomes. The esophagus is associated with an extensive submucosal lymphatic drainage network which contributes to locoregional recurrence of cancer. Adequate lymphadenectomy is crucial during esophagectomy. Proximal tumors often metastasize to upper mediastinal LN and middle esophageal tumors to mediastinal LN, and lower thoracic tumors often spread to abdominal LN [7,54].

The existing literature varies as to differences in efficacy of LN dissection between RAMIE and other surgical approaches. Some meta-analyses did not identify a significant difference in number of harvested LNs between RAMIE and MIE [36,55,56]. The ROBOT trial, published in 2019, published results of comparable LN harvest between RAMIE and OE [29]. On the other hand, a substantial quantity of recent literature supports the claim that RAMIE improves LN dissection precision and harvest [48,57,58,59,60]. Deng et al. documented a significantly increased total (21.9 versus 17.8) and abdominal-specific (10.8 versus 7.7) LN yield in RAMIE compared to MIE [45]. Ruurda et al. reported RAMIE LN harvest of 18–38 nodes versus 15–22 in MIE [61]. Meta-analysis by both Zhou et al. and Zhang et al. reported significantly increased LN harvest in RAMIE than MIE [32,34]. The 2023 meta-analysis by Zhang et al. also reported specifically that total, mediastinal-specific, and abdominal-specific LN harvest was increased in RAMIE [32]. Similarly, the aforementioned meta-analyses identified increased LN yield in RAMIE-MK as compared to RAMIE-IL, possibly attributed to improved mediastinal nodal dissection [32,34].

Further, RAMIE is suggested to improve lymphadenectomy surrounding the recurrent laryngeal nerves (RLNs) in particular [7]. The REVATE Trial noted a left RLN dissection rate of 88.3% in RAMIE and 69% in VATS—a statistically significant difference [27]. The RAMIE trial echoed these findings, noting a left RLN dissection rate of 79.5% in RAMIE and 67.6% in MIE (*p* = 0.001) [28]. These left RLN findings were concordant in various studies [37,51]. LN harvest surrounding the right RLN was also found to be significantly higher in RAMIE compared to MIE [37,45,51,58]. These findings may be attributed to improved maneuverability in the upper mediastinum and cervical region. Additionally, the literature suggests thoracic duct (TD) LN dissection improves overall lymphadenectomy yield and may afford an advantage in locoregional disease control; however, studies do not exist when comparing TD lymphadenectomy in RAMIE versus other approaches [7,58].

Overall, RAMIE purports equivalent if not superior LN harvest compared to MIE and OE. RAMIE allows for increased surgeon independence and more accurate and precise lymphadenectomy, especially in the setting of neoadjuvant therapy having been administered, with no increased morbidity from lymphadenectomy [6,51,58]. RAMIE does not confer an oncologically inferior LN dissection. High-quality lymphadenectomy with adequate LN harvest is a surrogate for long-term outcomes and survival. The ongoing ROBOT-2 Trial will hopefully provide the highest quality of evidence and shed clarity regarding LN retrieval in RAMIE [62].

#### 4.1.3. Operative Time

RAMIE yields longer operative times than OE. When compared to OE, RAMIE consistently incurs longer operating room times [33,56,63,64]. One study found a difference of 360 versus 306 min for RAMIE and OE, respectively (with a mean of an additional 69.5 min for RAMIE), while another cited a difference of 667 min versus 480 min on average for RAMIE and OE [33,58]. Meta-analyses estimated the difference at an additional 130 min for MIE and 150 min for RAMIE when compared to OE, and the randomized ROBOT trial calculated mean operating time at 170 min versus 134 min for RAMIE and OE [29,65]. The variations in average operative times likely reflect differences in surgical technique, study design, quality of evidence, and the time period of data collection, particularly with the advent of robotic esophagectomy.

The literature varies regarding the trend in operative times when investigating RAMIE versus MIE. When compared to MIE, some studies failed to find a significant difference for operative time between approaches, while others demonstrated significantly increased operative times for RAMIE—with RAMIE requiring an average of 33 additional minutes—than MIE [37,52,53]. The most recent and highest-quality evidence comes from the randomized controlled RAMIE trial published by Yang et al.: RAMIE incurred significantly shorter operation time than MIE (203.8 versus 244.9 min, *p* < 0.001) [28]. These results reflect great variation in RAMIE operative time based on surgeon proficiency. Much of the initial increased operative time with RAMIE is derived from docking, undocking, and repositioning. Additionally, a learning curve exists for performing the actual procedure. Learning curves vary based on surgeon level of credentialing and institutional practices for training—such as case volume thresholds for independent practice and structured robotic training programs. Despite the inconsistencies, studies have attempted to quantify case thresholds defining proficiency in RAMIE. Sarkaria et al. report a stepwise pathway to attain proficiency in RAMIE where pre-program cadaveric practice and on-console expert consultation are required training elements [19]. The literature suggests an average learning curve of 20 to 30 cases for RAMIE [19,33,37,58,66]. The main metric to determine learning curve is operative time. The German da Vinci Xi Registry Trial was able to demonstrate a decrease in average operating time as follows: 488 min for 0 to 15 cases, 402 min for 31–45 cases, and 349 min for 60 or more cases. Specifically, experienced surgeons were able to diminish their operative time by 4.8% after their 16th RAMIE [23]. In this context, the results of the RAMIE trial are congruent as this trial only included proficient surgeons beyond the inflection point of the learning curve. Operative time is an important outcome measure, and prolonged procedures requiring single-lung ventilation may increase the risk of postoperative pulmonary complications. Further, operative time represents a hospital quality metric, and prolonged operative times incur additional costs.

### 4.2. Postoperative Outcomes

Key comparative postoperative outcomes include overall, pulmonary, and cardiac complication rates; anastomotic and chyle leak rates; rate of RLN injury; rate of SSI; and length-of-stay (LOS) (Table 2).

#### 4.2.1. General

RAMIE is associated with equivalent rates of overall postoperative complications compared with MIE [44]. The RAMIE trial reported similar rates of both overall and major complications between the two approaches [5]. Furthermore, four recent meta-analyses concluded that overall complication rates for RAMIE and MIE were comparable, with no statistically significant differences [34,40,41,43]. Only one meta-analysis by Angeramo et al. compared RAMIE-IL to MIE and noted significantly lower postoperative morbidity with RAMIE (30% versus 40%, respectively; *p* < 0.001) [35]. This may represent an outlier finding. When evaluating RAMIE and MIE, consensus statements agreed that overall rates of postoperative complications are comparable [69].

In general, RAMIE was found to have improved postoperative outcomes compared to OE. This finding was demonstrated in the ROBOT trial, which found complication rates of 59% and 80%, respectively (*p* = 0.02) [29]. A meta-analysis by Esagian et al. found a difference in overall complications between RAMIE and MIE, although the trend did not quite reach statistical significance [33]. Another meta-analysis by Siaw-Acheampong et al. published similar findings, with a statistically significant difference between RAMIE and OE (*p* < 0.001) [42]. Mederos et al. further corroborated superiority of RAMIE to OE when comparing overall postoperative complications [40]. Van Kooten and colleagues specified patient-related factors that are prognostic indicators for major postoperative complications. The study identified male sex, cardiac comorbidity, and diabetes as factors significantly associated with major complications. A time interval between neoadjuvant therapy and surgery of less than 8 weeks was associated with fewer major complications [70].

#### 4.2.2. Pulmonary Complications

RAMIE has comparably lower rates of pneumonia and overall postoperative pulmonary complications to MIE and OE [42]. Previous meta-analyses comparing RAMIE to MIE have also demonstrated reduced pneumonia rates in RAMIE [32,34,35,39,41]. The ROBOT randomized trial and meta-analysis by Esagian et al. also found RAMIE to be superior to OE in postoperative pneumonia rates [29,33]. This meta-analysis specifically reported a trend of decreased acute respiratory distress syndrome (ARDS) rate after RAMIE than OE, although statistical significance was not reached [33]. Post-RAMIE pneumonia rates are estimated between 7–19.5% depending on surgical center and other factors [23,71].

When evaluating overall post-esophagectomy pulmonary complications, RAMIE ranked equivalent to MIE but superior to OE. The RAMIE trial cites pulmonary complication rates of 13.8% in RAMIE and 14.7% in MIE [28]. Meta-analyses estimated postoperative pulmonary complications for RAMIE between 18 and 20% and after MIE between 11 and 22%, with an overall lower risk associated with RAMIE (relative difference −0.06) [30,31,40]. Precise dissection and preservation of parasympathetic pulmonary innervation through sparing of vagal nerve branches is one proposed hypothesis for the rate of decreased pulmonary complications with RAMIE compared to OE [72]. The ROBOT trial found pulmonary complication rates of 32% after RAMIE compared to 58% in OE (*p* = 0.005), whereas the Esagian et al. meta-analysis identified rates of 14.29% after RAMIE compared to 25.32% after OE (*p* < 0.001), respectively [29,33]. Consensus statements report that 89% of experts agree RAMIE reduces the incidence of postoperative pulmonary complications compared with OE [69].

#### 4.2.3. Anastomotic Leak

The Esophageal Complications Consensus Group (ECCG) defines an anastomotic leak as a “full-thickness gastrointestinal defect involving the esophagus, anastomosis, staple line, or conduit” [72]. Anastomotic leak can result in mediastinitis and other sequelae. Anastomotic leak rates vary broadly between centers and among surgeons [67,73]. The literature varies but comprehensive estimates of 2–26% are quoted for post-esophagectomy anastomotic leak rates [58,70,71,72]. Extensive literature exists comparing anastomotic leak rates between RAMIE, MIE, and OE with no distinguishable differences [35,37]. Meta-analyses by Manigrasso et al. (evaluating 3482 procedures from 18 studies; *p* = 0.612), Mederos et al. (incorporating data from eight studies; relative difference of 0.0), Zheng et al. (evaluating 1352 patients from 11 studies; OR 1.13, *p* = 0.52), Angeramo et al. (OR 0.85, *p* = 0.22), Perry et al. (evaluating 10,002 patients; RR 1.23, *p* = 0.44), and Patton et al. (evaluating 2126 patients from seven randomized trials; OR 0.98) all found comparable rates of anastomotic leak between RAMIE and MIE [30,31,35,39,40,41]. The Zheng et al. meta-analysis found no difference in leak rates for cervical anastomoses between RAMIE and MIE, while the same was true for thoracic anastomoses between RAMIE and MIE [41]. When comparing RAMIE to OE, Esagian et al. identified a 6% leak rate for both RAMIE and OE (OR 0.93, *p* = 0.76), and Patton et al. identified an odds ratio of 0.7 with no statistically significant difference when comparing RAMIE to OE [31,33]. Randomized controlled trials confirmed these findings when comparing RAMIE to MIE and OE. The RAMIE trial found a 12.2% anastomotic leak rate in RAMIE and 11.3% in MIE (*p* = 0.801), while the ROBOT trial calculated a 22% anastomotic leak rate in RAMIE and 20% in OE (*p* = 0.57) [28,29].

Certain patient-specific factors have been identified as contributing to increased chance of anastomotic leak. Van Kooten et al. investigated patient-related prognostic factors and found that the following elements significantly influence postoperative anastomotic leak rate: (1) renal disease (OR 3.02), (2) increased hemoglobin A1c (OR 2.14), (3) vascular comorbidity (OR 1.53), (4) ASA score ≥ III (OR 1.49), (5) adenocarcinoma histology (OR 1.45), (6) diabetes (OR 1.40), (7) pulmonary comorbidity (OR 1.32), (8) hypertension (OR 1.26), (9) cardiac comorbidity (OR 1.24), and (10) male sex (OR 1.20). Neoadjuvant chemotherapy was a negative predictor for anastomotic leak (OR 0.88, *p* = 0.04) [70].

#### 4.2.4. RLN Injury

The RLN is susceptible to injury by stretch, compression, devascularization, and thermal damage during esophagectomy. RLN injury can lead to vocal cord dysfunction or paralysis, increasing the risk of pulmonary complications and additional sequelae. Improved visualization of the nerve with high-definition, magnified robotic cameras is thought to reduce RLN injuries. However, data suggest RAMIE is likely equivalent to MIE in protecting the RLNs. Multiple meta-analyses failed to find a significant difference in RLN palsy rates between RAMIE and MIE [30,33,37,39,40]. In these meta-analyses, the rate of vocal cord palsy or paralysis is estimated to be between 8.94 and 14% for RAMIE, approximately 7.63% for MIE, and approximately 10.41% for OE [30,33,72]. The randomized ROBOT trial failed to identify a difference in RLN injury between RAMIE and OE (*p* = 0.78), while the randomized RAMIE trial identified a higher, not statistically significant, rate of vocal cord paralysis in RAMIE compared to MIE (32.6% versus 27.1%) [28,29]. Alternatively, the REVATE trial discovered a lower RLN palsy rate at one-week post-esophagectomy for RAMIE compared to MIE and concluded that RAMIE allows for improved RLN nodal dissection and decreased rates of injury [27]. A few retrospective analyses and meta-analyses identified similar findings, recognizing a significantly lower rate of RLN injury with RAMIE [38,41,43]. Consensus statements show that only 67% of experts agreed RAMIE reduces the incidence of RLN injury, likely due to variations in surgeon technique despite technically improved dissection with the robotic surgical system [69].

#### 4.2.5. Cardiac Complications

RAMIE is associated with significantly decreased postoperative cardiac complications than MIE and OE [29,34,42]. Overall post-RAMIE cardiac complications are estimated at 0.56–15.74% [30,31].

Post-RAMIE atrial fibrillation is estimated at 6.79–22% [29,33]. RAMIE is associated with significantly decreased postoperative atrial fibrillation compared to OE [33,49]. Additionally, RAMIE is associated with significantly lower postoperative vasopressor requirements compared to MIE and OE [49].

#### 4.2.6. Chyle Leak

There is no significant difference in the rate of chylothorax after RAMIE when compared to MIE or OE [37,39,41,42]. Meta-analyses estimate chyle leak rates are 2.82–5.39% in RAMIE, 3.84% in MIE, and 3.01% in OE [30,42].

#### 4.2.7. Surgical Site Infection

The incidence of post-RAMIE surgical site infection (SSI) is equivalent to that of MIE. The incidence of post-RAMIE SSI is statistically superior to that of OE [39,41,42]. Meta-analyses estimate incidence of post-RAMIE SSI at 1.25–2.53%, while estimates are 3.18% for MIE and 5.95% for OE cohorts [30,42].

#### 4.2.8. Length-of-Stay and Recovery and Quality-of-Life

Available evidence on the association between RAMIE and hospital LOS remains conflicting. Generally, RAMIE and MIE result in reduced LOS compared to OE [33,42]. A large meta-analysis by Perry et al. identified a significant difference between RAMIE and MIE with a weighted mean difference of −3.03 days (*p* < 0.001) [30]. Significantly decreased hospital LOS for McKeown RAMIE versus MIE was also identified (weighted mean difference of −1.05 days) [34,72]. In meta-analysis by Esagian et al. and Siaw-Acheampong et al., RAMIE was associated with significantly decreased overall hospital length-of-stay (LOS) (weighted mean difference [WMD] of −9.22 days, *p* < 0.001 and WMD of −2.98 days, *p* < 0.001, respectively) compared to OE [33,42]. Other studies failed to detect this difference and reported comparable LOS between RAMIE, MIE, and OE [31,32,35,39,40,43]. In particular, the RAMIE trial did not find a significant difference in length of ICU stay (*p* = 0.990), readmission rates to the ICU (*p* = 0.815), or median length of hospital stay (*p* = 0.311) between RAMIE and MIE groups [28]. The meta-analysis by Siaw-Acheampong et al. did not find a difference in LOS between RAMIE and MIE as well (*p* = 0.371) [42]. The meta-analysis by Esagian et al. failed to identify a difference in hospital LOS between RAMIE and OE cohorts [33]. Overall, meta-analysis by Patel et al. estimated a total length of hospitalization for RAMIE all-comers at 15.2 days [71].

RAMIE has also been associated with more efficient postoperative functional recovery and improved quality-of-life than OE [6,7,72,74,75]. The ROBOT trial defined functional recovery as removal of thoracostomy tubes, absence of intravenous fluid requirements, tolerance of solid oral intake, independent mobilization, and adequate pain control on oral analgesics. The trial demonstrated a significant difference in recovery at postoperative day 14, with 70% of patients in the RAMIE group achieving the metric compared to 51% in the OE group (*p* = 0.038). The ROBOT trial also noted a significantly improved QOL at discharge (*p* = 0.02) and at six weeks post-discharge in the RAMIE group compared to the OE group (*p* = 0.03) [29]. RAMIE also results in decreased postoperative pain compared to OE [6,50]. Sarkaria et al. identified a lower Brief Pain Inventory (BPI) interference with life score in RAMIE patients compared to OE patients (*p* = 0.004) [50], findings that were sustained postoperatively out to two years [68]. Vimolratana et al. identified higher Functional Assessment of Cancer Therapy-Esophageal (FACT-E) after RAMIE than OE, and similarly lower BPI scores for RAMIE patients [68]. Less time in the ICU, decreased overall complication rate and hospital stay, and diminished postoperative pain all contribute to improved recovery in RAMIE in comparison to OE.

### 4.3. Oncologic Outcomes

Oncologic outcomes assessed in literature comparisons include R0 resection rate, recurrence-free and disease-free survival, and overall survival (Table 3).

#### Recurrence and Survival

RAMIE shows similar or improved rates of R0 resection and disease-free survival compared to MIE and OE [29,49,76]. Data demonstrate comparable or superior rates of R0 resection, defined as no macroscopic or microscopic evidence of cancer disease remaining at surgical pathology margins [28,77]. The RAMIE trial found a similar R0 resection rate between RAMIE and MIE [72]. Meta-analyses also concluded no difference exists between R0 resection rates between RAMIE and MIE or OE [32,33,43,44,46,60]. Only three meta-analyses—Siaw-Acheampong et al. (2020), Angeramo et al. (2021), and Manigrasso et al. (2021)—found RAMIE to be superior in terms of R0 resection when compared to MIE [35,39,42]. The 2010–2016 National Cancer Database (NCDB) review of RAMIE and MIE concluded RAMIE to have significantly lower rates of positive margins than MIE [6]. Rates of R0 resection in RAMIE are estimated at 95–98% [42,71,78].

Recurrence- and disease-free survival outcomes are also comparable between RAMIE, MIE, and OE [32,40,49]. Van der Sluis et al. found a median disease-free survival (DFS) of 23 months while a meta-analysis estimated median time of recurrence at 15 months for RAMIE and 9 months for MIE (*p* = 0.04) [76]. Yun et al. reported a 1-year DFS of 54.4% for RAMIE. At 21 months, distant and altogether recurrence rates after RAMIE and MIE were calculated via meta-analysis to be 11.8–14.9% and 10.2–25.5%, respectively, with no significant difference [49]. Locoregional-specific recurrence rates were estimated to be 3.5% for RAMIE and 3.9% for MIE in the same time frame [40]. The ROBOT trial stated comparable oncologic outcomes between RAMIE and MIE at 40 weeks post-esophagectomy [29]. At five-years postoperatively, van der Sluis et al. reported a five-year recurrence rate of 49.5% in RAMIE with locoregional-specific recurrence in 6%, systemic recurrence in 30%, and combined recurrence in 14% of patients [76]. Park et al. exhibited similar DFS rates between RAMIE (88%) and MIE (74%) at five years postoperatively (*p* = 0.10) [77]. Consensus statements indicate that 67% of experts agreed RAMIE provides better local control of tumor recurrence compared with MIE [69].

RAMIE incurs the same short-term mortality rates as MIE and OE and has comparable or superior long-term survival. Multiple studies have cited early survival at 30 and 90 days to be comparable among modalities [32,33,37,39,42,44,58,69]. Meta-analyses by Yun et al., Yerokun et al., and Siaw-Acheampong et al. identified better overall survival (OS) with RAMIE [42,47,49]. The paper by Siaw-Acheampong et al. quotes improved one-year OS for RAMIE compared to MIE and improved five-year OS for RAMIE compared to OE, whereas Yerokun et al. note a significantly higher three-year OS for RAMIE compared to MIE (84% versus 56%, *p* = 0.034) [42,47]. Still, the majority of research declares that RAMIE, MIE, and OE have a similar OS [29,32,33,39,46]. Weksler et al. described a median OS of 48 months among RAMIE patients and 44 months among OE patients, with no significant difference (*p* = 0.53) [46]. A propensity-matched study reported slightly longer median survival after RAMIE at 58.8 months without a significant difference in median OS of 45.9 months after MIE [40]. Van der Sluis et al. reported a median OS of 29 months [76]. Yun et al. reported a one-year overall survival (OS) of 95.1% after RAMIE compared with 85.6% after OE, and a three-year OS of 81.7% after RAMIE versus 73.7% after OE, with no significant difference between the groups [49]. Van der Sluis and colleagues found a five-year OS rate of 42% after RAMIE [76]. Overall, RAMIE is as oncologically effective as MIE and OE, with comparable incidence of recurrence and equivalent long-term survival.

## 5. Challenges and Limitations

### 5.1. Cost

RAMIE is performed utilizing a robotic surgical system, with the da Vinci Robotic System (Intuitive Surgical, Inc., Sunnyvale, CA, USA) most commonly used worldwide [79]. However, additional robotic surgical platform options are rapidly emerging internationally. Robotic surgical systems incur a high initial investment cost between 1.5 to 2.5 million dollars and typically include the surgeon console, patient cart, and a vision cart [24,63,80]. Ongoing investments additionally include a yearly service contract of 100,000 to 190,000 US dollars [81]. Significantly elevated acquisition costs, equipment purchasing and maintenance costs, and prolonged duration of robotic procedures garner increased surgical costs of RAMIE when compared to MIE or open procedures [24,63,82,83].

Despite higher operative costs, the literature suggests the overall cost for RAMIE is equivalent to MIE or OE approaches to esophagectomy [82,84]. Cost analysis of the ROBOT trial found that postoperative care, hospitalization expenses, and overall costs were comparable between robotic esophagectomy and other minimally invasive or open techniques, notwithstanding significantly greater surgical costs associated with the use of a robot [24]. Additional studies have cited fewer complications, and shorter intensive care unit (ICU) and hospital LOS, and thus lower hospital care costs, as factors that equalize total cost when comparing RAMIE to MIE [24,82]. When evaluating additional financial expenses, no significant difference was identified in the cost of anesthesiology and cardiology care, intensive care unit or ward stays, laboratory tests, dialysis, endoscopy procedures, diagnostic procedures, or physiotherapy for RAMIE or MIE [82]. Overall, RAMIE appears financially comparable to MIE and OE approaches. Further, conclusions of the ROBOT trial noted that the greater cost incurred during operation is minimal in context of the overall surgical and oncologic treatment for a patient’s esophageal cancer [24]. More data are needed to elucidate the financial impact of RAMIE.

### 5.2. Accessibility and Sustainability

As of 2017, at least 4149 robotic surgical systems had been installed worldwide, with over 50% installed within the United States of America [63]. The worldwide number of systems installed by the end of 2025 had skyrocketed to 10,763 [85]. However, access to robotic surgical systems and instruments remains a challenge in remote and low-resource settings. Equipment for maintenance, such as sterilization of reusable instruments, may also be of limited availability in certain settings [82]. Eliminating monopolies on surgical robotic systems in favor of a free or competing markets may decrease required investment costs, allowing for increased global access [30].

Further, the environmental impact of robotic esophageal procedures has not been studied. The literature suggests robotic procedures impose a significant burden on environmental sustainability [86,87,88,89,90]. Strategies to mitigate environmental impact may include minimizing packaging materials, utilizing reusable materials, advancing technologies regarding sterilization of reusable instruments, and increasing operating personnel awareness regarding attention to limiting instrument use [24]. More research performed in high-volume centers is necessary to evaluate the economic and environmental impact of RAMIE and MIE approaches [80,82]. Hospital administrations and policy-makers may inform advancements in the field of robotic surgery that are sustainable and economically feasible around the globe [24,80].

### 5.3. Technical Variability

Standardization of RAMIE is challenging due to differences in patient population and tumor features as well as differences in robotic use across phases and institutions. Techniques in esophageal surgery are not consistent globally. The 2023 Upper Gastrointestinal International Robotic Association (UGIRA) registry included 38 centers performing RAMIE [91]. High-volume centers from Europe, Asia, North America, and South America were included in this registry, highlighting the geographic disparities in the performance of RAMIE. Within centers that perform RAMIE, variability exists among providers. RAMIE as a surgical approach, as compared to MIE or OE, is becoming increasingly preferred over time. An analysis of 10,607 esophagectomies in the Society of Thoracic Surgeons General Thoracic Surgery Database from 2015 to 2019 showed a decrease in the OE rate from 58% to 42%, while the rate of MIE increased from 21% to 40% and RAMIE increased from 1% to 18% [92]. A 2025 systematic review incorporating data from 2729 cases in sixteen randomized controlled trials found that OE was performed in 50.6% of cases, while MIE, hybrid surgery, and RAMIE were performed in 31.4%, 5.4%, and 12.6% of cases, respectively [93]. Continued research, informed evidence-based practice, and time may elicit the adoption of standardized esophagectomy practices.

## 6. Future Directions

### 6.1. Artificial Intelligence (AI) and Surgical Automation

Artificial intelligence (AI) and surgical automation are emerging adjuncts to RAMIE.

High-quality video platforms generate standardized digital data that can be analyzed using “computer vision” and deep learning algorithms to identify operative phases, surgical instruments, and key anatomy [24,94]. Takeuchi et al. demonstrated that AI could correctly classify RAMIE operative phases in 84% of cases using annotated intraoperative videos [95]. These technologies may enhance surgical education through augmented or virtual reality simulation, automated performance assessment, personalized training modules and feedback, and remote mentoring [91,94]. AI-assisted analysis of intraoperative video may also provide objective skill evaluation and facilitate mastery of complex robotic techniques [24,94]. AI can be utilized to effectively and efficiently train surgeons and reduce complications.

Beyond training, AI has potential applications throughout the perioperative period. Predictive models may assist with patient selection, preoperative planning, three-dimensional anatomical modeling, and decision support [24,94]. Computer-aided anatomy recognition systems have demonstrated the ability to identify major thoracic structures during RAMIE with accuracy comparable to expert surgeons, potentially improving operative safety and reducing the learning curve [94]. AI may further augment robotic precision through motion compensation, automated monitoring, and prediction of intra- or postoperative complications based on operative video analysis [24,91,94]. Further, automated monitoring and documentation provided by AI may detect physiologic alterations prior to human detection and allow for earlier intervention [94]. Such monitoring may be accomplished remotely as well, which may generate global collaborate networks of gastrointestinal and thoracic surgeons. However, these technologies remain in early development and depend on high-quality annotated surgical datasets [24,94]. Large-scale validation studies and prospective trials are still needed before widespread adoption in robotic esophageal surgery.

### 6.2. Intraoperative Imaging Adjuncts

Intraoperative imaging adjuncts have been utilized in RAMIE. Sarkaria et al. reported the use of near-infrared fluorescence imaging fluorescence angiography, specifically with indocyanine green (ICG), to the vascular supply during esophagectomy [96]. Other studies similarly report the use of imaging adjuncts to evaluate anatomy and vascular arcades during RAMIE [97]. Further, ongoing developments for tumor localization include tumor-targeted tracers with intraoperative molecular imaging [98] and the expansion in use of folate receptor-targeted fluorescent agents, such as pafolacianine, from lung to esophageal cancer localization [99]. Investigation in this realm is ongoing, and maturation of results is anticipated.

### 6.3. Emerging Robotic Systems

Another emerging forefront in RAMIE is the use of new, multi-arm robotic surgical systems, and the use of single-port (SP) methods. The SP technique has been implemented for anatomic lung resection procedures, with the goal of reducing postoperative pain from port site intercostal nerve compression [100]. Yet the degree of complexity associated with esophagectomy, and the posterior mediastinal location of the esophagus with a crowded working space, make application of single-port RAMIE more challenging. For this reason, the learning curve for SP RAMIE is even steeper than for traditional RAMIE [100]. A uniportal robotic-assisted esophagectomy thoracic approach is described by Dmitrii and Pavel: a 3–4 cm incision is made in the fifth intercostal space between the mid- and posterior axillary lines; a wound protector is placed; and, a flexible high-definition thoracoscope and traditional instruments are inserted and utilized [101]. The SP robot also enables a cervical approach under mediastinoscopic visualization. Avoidance of operating within the pleural cavity in this manner reduces pulmonary complications and allows treatment of exceptionally proximal esophageal tumors [24]. This combination of advantages allows for surgical management of patients who might not tolerate single-lung ventilation or who were deemed previously unresectable.

## 7. Conclusions

Intraoperative methods for assessment of surgeon performance include EBL, number of harvested lymph nodes, and operative time. Crucial postoperative outcomes include overall morbidity, rates of cardiac and pulmonary complications, rate of RLN injury, anastomotic and chyle leak, recovery time, and oncologic recurrence and survival. Adequate preoperative evaluation and a standardized surgical technique may improve post-esophagectomy outcomes. RAMIE is a vastly different version of esophagectomy when compared to the historical Torek procedures in 1913. Yet, room for development exists for RAMIE. The future consists of improving accessibility, cost, and sustainability of surgical robots while incorporating AI and advanced technologies into RAMIE.

## Figures and Tables

**Table 1:** Intraoperative metrics.

Study	Year	Design	N (RAMIE/MIE/OE)	Comparison	Estimated Blood Loss	Lymph Node Harvest	Operative Time
Chao [27]	2024	RCT	103/100/–	RAMIE vs. MIE	No difference (NS)	Higher (*p* = 0.035)	Shorter (*p* < 0.001)
Yang [28]	2021	RCT	181/177/–	RAMIE vs. MIE	No difference (NS)	Higher left RLN dissection success (*p* = 0.001)	Shorter (*p* < 0.001)
van der Sluis [29]	2019	RCT	54/–/55	RAMIE vs. OE	Lower (*p* < 0.001)	No difference (*p* = 0.41)	Longer (*p* < 0.001)
Perry [30]	2024	Meta-analysis	5205/12,982/–	RAMIE vs. MIE	Lower (*p* < 0.00001)	Higher total (*p* < 0.0001); higher left RLN (*p* = 0.03)	Longer (*p* = 0.004)
Patton [31]	2024	Network meta-analysis	235/361/350 (+117 HE)	RAMIE vs. MIE/OE	No difference (NS)	NR	NR
Zhang [32]	2022	Meta-analysis	1418/1514/–	RAMIE vs. MIE	No difference (NS)	Higher (*p* < 0.001)	No difference (NS)
Esagian [33]	2022	Meta-analysis	674/–/1303	RAMIE vs. OE	Lower (*p* < 0.001)	No difference (NS)	Longer (*p* < 0.001)
Zhou [34]	2022	Meta-analysis	863/862/–	RAMIE vs. MIE	NR	Higher (reported significant)	NR
Angeramo [35]	2021	Meta-analysis	974/5275/–	RAMIE vs. MIE	Lower (*p* < 0.0001)	No difference (NS)	Longer (*p* < 0.0001)
Chen [36]	2021	Meta-analysis	2306/2408/–	RAMIE vs. MIE	No difference (NS)	Higher total (*p* < 0.001); higher left RLN (*p* < 0.001)	No difference (NS)
Huang [37]	2021	Meta-analysis	1921/1917/–	RAMIE vs. MIE	Lower (*p* = 0.008)	No difference (NS)	Longer (*p* = 0.01)
Li [38]	2021	Meta-analysis	866/883/–	RAMIE vs. MIE	Lower (*p* = 0.007)	Higher total (*p* = 0.011); higher abdominal (*p* = 0.009); higher RLN (*p* < 0.001)	NR
Manigrasso [39]	2021	Meta-analysis	3832/11,779/4485	RAMIE vs. MIE/OE	Lower vs. OE (*p* = 0.001)	Higher (*p* < 0.0001 vs. OE; *p* = 0.001 vs. MIE)	Longer (*p* < 0.05)
Mederos [40]	2021	Systematic review/meta-analysis	9355 total	RAMIE vs. MIE/OE	Lower vs. OE (*p* < 0.001); NS vs. MIE	Higher vs. OE (*p* = 0.005); NS vs. MIE	Longer (*p* < 0.001)
Zheng [41]	2021	Meta-analysis	1435/1452/–	RAMIE vs. MIE	NR	NR	Longer (reported significant)
Siaw-Acheampong [42]	2020	Network meta-analysis	917/9558/17,824	RAMIE vs. MIE/OE	Lower vs. OE (ranked best)	Higher (ranked best)	Longer (ranked)
Jin [43]	2019	Meta-analysis	931/931/–	RAMIE vs. MIE	Lower (*p* = 0.0075)	No difference (NS)	No difference (*p* = 0.2402)
Nishigori [44]	2024	PSM cohort	546/546/–	RAMIE vs. MIE	Higher (*p* = 0.02)	NR	Longer (*p* < 0.001)
Deng [45]	2018	PSM cohort	52/52/–	RAMIE vs. MIE	No difference (*p* = 0.158)	Higher total (*p* = 0.006); higher abdominal (*p* = 0.042); higher left RLN (*p* = 0.033)	Longer (*p* < 0.001)
Weksler [46]	2017	Database cohort	581/2379/6257	RAMIE/MIE/OE	NR	No difference (NS)	NR
Yerokun [47]	2016	Database cohort	231/1308/2958	RAMIE/MIE vs. OE	NR	Higher vs. OE (*p* = 0.016)	NR
Kooij (UGIRA) [48]	2024	International registry cohort	608 RAMIE	Contemporary RAMIE outcomes	Low median EBL reported	High nodal yield reported	Benchmark operative metrics reported
Yun [49]	2019	Cohort	130/–/241	RAMIE vs. OE	No difference (*p* = 0.251)	No difference (*p* = 0.571)	Longer (*p* = 0.001)
Sarkaria [50]	2019	Prospective cohort	64/–/106	RAMIE vs. OE	Lower (*p* < 0.001)	Higher (*p* = 0.045)	Longer (*p* < 0.001)
Motoyama [51]	2019	Cohort	21/38/–	RAMIE vs. MIE	No difference thoracic (*p* = 0.168)	Higher left RLN ratio (*p* = 0.048)	No difference (*p* = 0.601 thoracic; *p* = 0.363 total)
Weksler [52]	2011	Cohort	11/26/–	RAMIE vs. MIE	No difference (NS)	No difference (NS)	No difference (NS)
Suda [53]	2012	Prospective cohort	16/20/–	RAMIE vs. MIE	No difference (*p* = 0.055 thoracic; *p* = 0.757 total)	No difference (*p* = 0.486 chest)	No difference (*p* = 0.506 thoracic)

Abbreviations: RAMIE = robot-assisted minimally invasive esophagectomy; MIE = minimally invasive esophagectomy; OE = open esophagectomy; HE = hybrid esophagectomy; LN = lymph node; PSM = propensity score matched; RCT = randomized controlled trial; NR = not reported; NS = not significant. Exact *p*-values are shown when extractable from the source article; “NS” indicates the source reported no statistically significant difference without providing an exact *p*-value.

**Table 2:** Postoperative outcomes.

Study	Year	Design	N (RAMIE/MIE/OE)	Comparison	Overall Complications	Pulmonary Complications	Cardiac Complications	Anastomotic Leak	RLN Injury	Chyle Leak	SSI	LOS
Chao [27]	2024	RCT	103/100/–	RAMIE vs. MIE	No difference (*p* = 0.548)	No difference (*p* = 0.933)	NR	No difference (*p* = 0.157)	Lower (*p* = 0.029 short-term; *p* = 0.003 permanent)	No difference (*p* = 0.364)	NR	No difference (*p* = 0.402)
Yang [28]	2021	RCT	181/177/–	RAMIE vs. MIE	No difference (*p* = 0.196)	No difference (*p* = 0.812)	NR	No difference (*p* = 0.801)	No difference (*p* = 0.258)	NR	NR	No difference (NS)
van der Sluis [29]	2019	RCT	54/–/55	RAMIE vs. OE	Lower (*p* = 0.02)	Lower (*p* = 0.005)	Lower (*p* = 0.006)	No difference (NS)	No difference (NS)	No difference (NS)	NR	No difference (NS)
Perry [30]	2024	Meta-analysis	5205/12,982/–	RAMIE vs. MIE	No difference (NS)	Lower (*p* = 0.001)	No difference (*p* = 0.88)	Higher in RAMIE (*p* = 0.0005)	No difference (*p* = 0.62)	No difference (*p* = 0.74)	No difference (*p* = 0.92)	Shorter (*p* < 0.0001)
Patton [31]	2024	Network meta-analysis	235/361/350 (+117 HE)	RAMIE vs. MIE/OE	No difference (NS)	Lower vs. OE (OR 3.63 for OE; *p* < 0.05)	No difference (NS)	No difference (NS)	NR	NR	NR	No difference (NS)
Zhang [32]	2022	Meta-analysis	1418/1514/–	RAMIE vs. MIE	No difference (*p* = 0.605)	Lower pneumonia (*p* = 0.01)	NR	No difference (*p* = 0.906)	No difference (*p* = 0.21)	No difference (NS)	NR	No difference (NS)
Esagian [33]	2022	Meta-analysis	674/–/1303	RAMIE vs. OE	No difference (NS)	Lower pulmonary (*p* < 0.001); lower pneumonia (*p* < 0.001)	Lower atrial fibrillation (*p* = 0.04)	No difference (NS)	No difference (NS)	No difference (NS)	Lower wound infection (*p* < 0.001)	Shorter (*p* < 0.001)
Zhou [34]	2022	Meta-analysis	863/862/–	RAMIE vs. MIE	No difference (NS)	Lower (*p* = 0.04)	NR	No difference (NS)	No difference (NS)	No difference (NS)	NR	Shorter (*p* < 0.0001)
Angeramo [35]	2021	Meta-analysis	974/5275/–	RAMIE vs. MIE	Lower (*p* < 0.001)	Lower pneumonia (*p* < 0.001)	NR	No difference (NS)	NR	NR	NR	No difference (NS)
Chen [36]	2021	Meta-analysis	2306/2408/–	RAMIE vs. MIE	No difference (NS)	Lower pneumonia (*p* = 0.036)	NR	No difference (*p* = 0.053)	No difference (*p* = 0.324/NS)	No difference (*p* = 0.161)	NR	No difference (NS)
Huang [37]	2021	Meta-analysis	1921/1917/–	RAMIE vs. MIE	No difference (NS)	Lower (*p* = 0.003)	No difference (*p* = 0.82)	No difference (*p* = 0.84)	No difference (*p* = 0.69)	No difference (*p* = 0.86)	No difference (*p* = 0.88)	No difference (NS)
Li [38]	2021	Meta-analysis	866/883/–	RAMIE vs. MIE	No difference (NS)	NR	NR	NR	Lower vocal cord palsy sensitivity analysis (*p* = 0.027)	NR	NR	NR
Manigrasso [39]	2021	Meta-analysis	3832/11,779/4485	RAMIE vs. MIE/OE	NR	Lower pneumonia vs. OE (*p* = 0.030)	NR	No difference (NS)	Lower vs. MIE (*p* = 0.003)	No difference (NS)	Lower wound infection vs. OE (*p* = 0.002)	NR
Mederos [40]	2021	Systematic review/meta-analysis	9355 total	RAMIE vs. MIE/OE	No difference (NS)	Lower vs. OE (*p* = 0.001); NS vs. MIE	NR	No difference (NS)	No difference (NS)	NR	NR	No difference (NS)
Zheng [41]	2021	Meta-analysis	1435/1452/–	RAMIE vs. MIE	No difference (NS)	Lower (reported significant)	NR	No difference (NS)	NR	NR	NR	NR
Siaw-Acheampong [42]	2020	Network meta-analysis	917/9558/17,824	RAMIE vs. MIE/OE	RAMIE ranked best	MIE best; RAMIE favorable	RAMIE best (cardiac)	NR	NR	NR	NR	RAMIE best
Jin [43]	2019	Meta-analysis	931/931/–	RAMIE vs. MIE	No difference (NS)	No difference (*p* = 0.95)	NR	No difference (*p* = 0.13)	Lower vocal cord palsy (*p* = 0.0447)	NR	NR	No difference (*p* = 0.1342)
Nishigori [44]	2024	PSM cohort	546/546/–	RAMIE vs. MIE	No difference (*p* = 0.52)	No difference (NS)	NR	No difference (NS)	Trend higher (*p* = 0.06)	NR	Lower deep SSI (reported)	Longer (*p* = 0.001)
Deng [45]	2018	PSM cohort	52/52/–	RAMIE vs. MIE	No difference major complications (*p* = 0.979)	No difference severe pneumonia (*p* = 0.878)	NR	No difference (*p* = 0.748)	No difference (*p* = 0.844)	NR	NR	No difference (*p* = 0.120)
Weksler [46]	2017	Database cohort	581/2379/6257	RAMIE/MIE/OE	NR	NR	NR	NR	NR	NR	NR	NR
Yerokun [47]	2016	Database cohort	231/1308/2958	RAMIE/MIE vs. OE	NR	NR	NR	NR	NR	NR	NR	Shorter vs. OE (*p* = 0.046)
Milone [67]	2025	Cohort	NR	RAMIE	Acceptable morbidity reported	NR	NR	Focused analysis of leak prevention/perfusion	NR	NR	NR	NR
Vimolratana [68]	2021	Cohort/QOL study	NR	RAMIE vs. OE/MIE	Improved postoperative recovery/QOL reported	NR	NR	NR	NR	NR	NR	Faster recovery/QOL benefit reported
Yun [49]	2019	Cohort	130/–/241	RAMIE vs. OE	No difference (NS)	Lower pneumonia (*p* = 0.035)	NR	NR	NR	NR	Wound problems lower long-term (*p* = 0.020)	NR
Sarkaria [50]	2019	Prospective cohort	64/–/106	RAMIE vs. OE	No difference (*p* > 0.95)	Lower (*p* = 0.014)	NR	No difference (*p* > 0.41)	NR	NR	Lower infectious (*p* = 0.029)	Shorter (*p* < 0.001)
Motoyama [51]	2019	Cohort	21/38/–	RAMIE vs. MIE	No difference (NS)	No difference (1.0)	NR	No difference (1.0)	Trend lower (*p* = 0.098)	No difference (1.0)	NR	NR
Weksler [52]	2011	Cohort	11/26/–	RAMIE vs. MIE	No difference (NS)	No difference (NS)	NR	No difference (NS)	No difference (NS)	NR	NR	No difference (NS)
Suda [53]	2012	Prospective cohort	16/20/–	RAMIE vs. MIE	No difference (*p* = 0.693)	No difference (NS)	No difference (*p* = 0.313)	Higher (*p* = 0.038)	Lower vocal cord palsy (*p* = 0.018); lower hoarseness (*p* = 0.015)	No difference (*p* = 0.597)	NR	No difference (*p* = 0.068)

Abbreviations: RAMIE = robot-assisted minimally invasive esophagectomy; MIE = minimally invasive esophagectomy; OE = open esophagectomy; LN = lymph node; RLN = recurrent laryngeal nerve; SSI = surgical site infection; LOS = length-of-stay; PSM = propensity score matched; RCT = randomized controlled trial; NR = not reported; NS = not significant. Exact *p*-values are shown when extractable from the source article; “NS” indicates the source reported no statistically significant difference without providing an exact *p*-value.

**Table 3:** Oncologic outcomes.

Study	Year	Design	N (RAMIE/MIE/OE)	Comparison	R0 Resection Rate	RFS/DFS	Overall Survival
Chao [27]	2024	RCT	103/100/–	RAMIE vs. MIE	No difference (NS)	NR	NR
Yang [28]	2021	RCT	181/177/–	RAMIE vs. MIE	No difference (NS)	NR	NR
van der Sluis [29]	2019	RCT	54/–/55	RAMIE vs. OE	No difference (NS)	No difference (NS)	No difference (NS)
Perry [30]	2024	Meta-analysis	5205/12,982/–	RAMIE vs. MIE	No difference (NS)	NR	No difference (NS)
Patton [31]	2024	Network meta-analysis	235/361/350 (+117 HE)	RAMIE vs. MIE/OE	No difference (NS)	No difference (NS)	No difference (NS)
Zhang [32]	2022	Meta-analysis	1418/1514/–	RAMIE vs. MIE	No difference (NS)	Higher 3-y DFS (*p* = 0.006)	No difference (NS)
Esagian [33]	2022	Meta-analysis	674/–/1303	RAMIE vs. OE	No difference (NS)	NR	No difference (NS)
Zhou [34]	2022	Meta-analysis	863/862/–	RAMIE vs. MIE	No difference (NS)	No difference (NS)	No difference (NS)
Angeramo [35]	2021	Meta-analysis	974/5275/–	RAMIE vs. MIE	No difference (NS)	NR	No difference (NS)
Chen [36]	2021	Meta-analysis	2306/2408/–	RAMIE vs. MIE	NR	NR	No difference (NS)
Huang [37]	2021	Meta-analysis	1921/1917/–	RAMIE vs. MIE	No difference (NS)	NR	No difference (*p* = 0.82)
Li [38]	2021	Meta-analysis	866/883/–	RAMIE vs. MIE	NR	No difference (NS)	No difference (NS)
Manigrasso [39]	2021	Meta-analysis	3832/11,779/4485	RAMIE vs. MIE/OE	Higher vs. OE (*p* = 0.043)	No difference (NS)	NR
Mederos [40]	2021	Systematic review/meta-analysis	9355 total	RAMIE vs. MIE/OE	NR	No difference/NR	No difference/NR
Zheng [41]	2021	Meta-analysis	1435/1452/–	RAMIE vs. MIE	NR	NR	NR
Siaw-Acheampong [42]	2020	Network meta-analysis	917/9558/17,824	RAMIE vs. MIE/OE	No difference (ranked)	NR	Mixed/NR
Jin [43]	2019	Meta-analysis	931/931/–	RAMIE vs. MIE	NR	NR	No difference (NS)
Nishigori [44]	2025	PSM cohort	546/546/–	RAMIE vs. MIE	Lower (*p* = 0.02)	NR	NR
Deng [45]	2018	PSM cohort	52/52/–	RAMIE vs. MIE	NR	NR	NR
Weksler [46]	2017	Database cohort	581/2379/6257	RAMIE/MIE/OE	NR	NR	No difference (*p* = 0.53 matched)
Yerokun [47]	2016	Database cohort	231/1308/2958	RAMIE/MIE vs. OE	No difference (*p* > 0.05)	NR	No difference (*p* > 0.05)
Kooij (UGIRA) [48]	2025	International registry cohort	608 RAMIE	Contemporary RAMIE outcomes	High R0 rate reported	NR	NR
Yun [49]	2019	Cohort	130/–/241	RAMIE vs. OE	No difference (*p* = 0.719)	No difference recurrence (*p* = 0.191); higher DFS (*p* = 0.006)	Higher OS (*p* = 0.001)
Sarkaria [50]	2019	Prospective cohort	64/–/106	RAMIE vs. OE	No difference (NS)	NR	NR
Motoyama [51]	2019	Cohort	21/38/–	RAMIE vs. MIE	NR	NR	NR
Weksler [52]	2011	Cohort	11/26/–	RAMIE vs. MIE	No difference (NS)	NR	NR
Suda [53]	2012	Prospective cohort	16/20/–	RAMIE vs. MIE	No difference (*p* = 0.586)	NR	NR

Abbreviations: RAMIE = robot-assisted minimally invasive esophagectomy; MIE = minimally invasive esophagectomy; OE = open esophagectomy; HE = hybrid esophagectomy; RFS = recurrence-free survival; DFS = disease-free survival; PSM = propensity score matched; RCT = randomized controlled trial; NR = not reported; NS = not significant. Exact *p*-values are shown when extractable from the source article; “NS” indicates the source reported no statistically significant difference without providing an exact *p*-value.

## Data Availability

No new data were created or analyzed in this study. Data sharing is not applicable to this article.
